# Evaluation of Three Quantitative Anti-SARS-CoV-2 Antibody Immunoassays

**DOI:** 10.1128/spectrum.01376-21

**Published:** 2021-12-22

**Authors:** Sabine Chapuy-Regaud, Marcel Miédougé, Florence Abravanel, Isabelle Da Silva, Marion Porcheron, Judith Fillaux, Chloé Diméglio, Jacques Izopet

**Affiliations:** a Department of Virology, CHU Purpan, Toulouse, France; b INFINITy (Institut Toulousain des Maladies Infectieuses et Inflammatoires) INSERM UMR1291/CNRS UMR5051/Université Toulouse III, CHU Purpan, Toulouse, France; c Department of Parasitology, CHU Purpan, Toulouse, France; Fundacio irsiCaixa

**Keywords:** SARS-CoV-2, immunoassay, binding antibodies, neutralizing antibodies, COVID

## Abstract

The severe acute respiratory syndrome coronavirus 2 (SARS-CoV-2) emerged in December 2019 and caused a dramatic pandemic. Serological assays are used to check for immunization and assess herd immunity. We evaluated commercially available assays designed to quantify antibodies directed to the SARS-CoV-2 Spike (S) antigen, either total (Wantaï SARS-CoV-2 Ab ELISA) or IgG (SARS-CoV-2 IgG II Quant on Alinity, Abbott, and Liaison SARS-CoV-2 TrimericS IgG, Diasorin). The specificities of the Wantaï, Alinity, and Liaison assays were evaluated using 100 prepandemic sera and were 98, 99, and 97%, respectively. The sensitivities of all three were around 100% when tested on 35 samples taken 15 to 35 days postinfection. They were less sensitive for 150 sera from late infections (>180 days). Using the first WHO international standard (NIBSC), we showed that the Wantai results were concordant with the NIBSC values, while Liaison and Alinity showed a proportional bias of 1.3 and 7, respectively. The results of the 3 immunoassays were significantly globally pairwise correlated and for late infection sera (*P* < 0.001). They were correlated for recent infection sera measured with Alinity and Liaison (*P* < 0.001). However, the Wantai results of recent infections were not correlated with those from Alinity or Liaison. All the immunoassay results were significantly correlated with the neutralizing antibody titers obtained using a live virus neutralization assay with the B1.160 SARS-CoV-2 strain. These assays will be useful once the protective anti-SARS-CoV-2 antibody titer has been determined.

**IMPORTANCE** Standardization and correlation with virus neutralization assays are critical points to compare the performance of serological assays designed to quantify anti-SARS-CoV-2 antibodies in order to identify their optimal use. We have evaluated three serological immunoassays based on the virus spike antigen that detect anti-SARS-CoV-2 antibodies: a microplate assay and two chemiluminescent assays performed with Alinity (Abbott) and Liaison (Diasorin) analysers. We used an in-house live virus neutralization assay and the first WHO international standard to assess the comparison. This study could be useful to determine guidelines on the use of serological results to manage vaccination and treatment with convalescent plasma or monoclonal antibodies.

## INTRODUCTION

The severe acute respiratory syndrome coronavirus 2 (SARS-CoV-2) emerged in Wuhan, China in December 2019 and caused a dramatic pandemic (1). Anti-SARS-CoV-2 antibodies are essential tools for managing and understanding how coronavirus disease 2019 (COVID-19) spreads through populations and for measuring herd immunity and individual immune response. Some studies have evaluated the correlation of antibodies measured in immunoassays with their neutralization capacity (2, 3). However, more and more commercial assays are available, and their relative performances must be evaluated. Indeed, serological assays differ in the immunoreactive antigen used, the class of antibodies detected, their ability to quantify antibodies, and their implementation on an automated device.

The initial assays, which were designed to detect antibodies against the nucleocapsid (N) or the spike (S) protein, had similar capacities for detecting anti-SARS-CoV-2 antibodies 2 weeks post-symptom onset (4–8). Neutralizing antibodies mainly target the receptor-binding domain (RBD) of the S protein (9). For this reason, most WHO-approved vaccines are based on the S protein, while some are inactivated vaccines (as listed at https://www.who.int/emergencies/diseases/novel-coronavirus-2019/covid-19-vaccines).

Vaccination poses several challenges for SARS-CoV-2 serology because one objective is to determine an antibody concentration that confers full protection against the virus. An ideal immunoassay must quantify the antibodies and provide a binding antibody titer that is correlated with the neutralizing antibody titer (10, 11). Serological assays must be able to also measure multiple immunoglobulin classes because the IgM is produced in the early response but does not persist for as long as IgG and IgA, which are long-lasting antibodies (12, 13). The relationship between analytical methods is also essential for the full evaluation of biomedical laboratory results. To this end, the UK National Institute for Biological Standards and Control (NIBSC) has prepared a reference control material (14).

This study evaluated the clinical performances and antibody quantifying capacity of three commercially available assays. We used the first NIBSC standard as a reference for anti-S antibodies (14, 15). The Wantaï SARS-CoV-2 antibody (Ab) enzyme-linked immunosorbent assay (ELISA) measures total anti-SARS-CoV-2 antibodies. The SARS-CoV-2 IgG II Quant used with Alinity analyzer (Abbott) and the LIAISON SARS-CoV-2 TrimericS IgG (Diasorin) are chemiluminescence immunoassays designed to measure anti-SARS-CoV-2 IgG on routine laboratory automated systems. We initially chose the Wantaï method to manage SARS-CoV-2 serologies in our clinical biology laboratory (Laboratory of Virology, Toulouse University Hospital) because this assay was one of the first and most performant available tests (16). The evaluation of the automatized methods (Alinity and Liaison) was further chosen given the ongoing use of these multiparametric devices for other serological analyzes. The Abbott and Liaison assays are the latest versions from these manufacturers and few comparative data are available. We also determined the correlation between the binding antibody titers measured by these immunoassays and neutralizing antibodies titrated using a live virus-based assay.

## RESULTS

### Immunoassay clinical performance.

**FIG 1:**
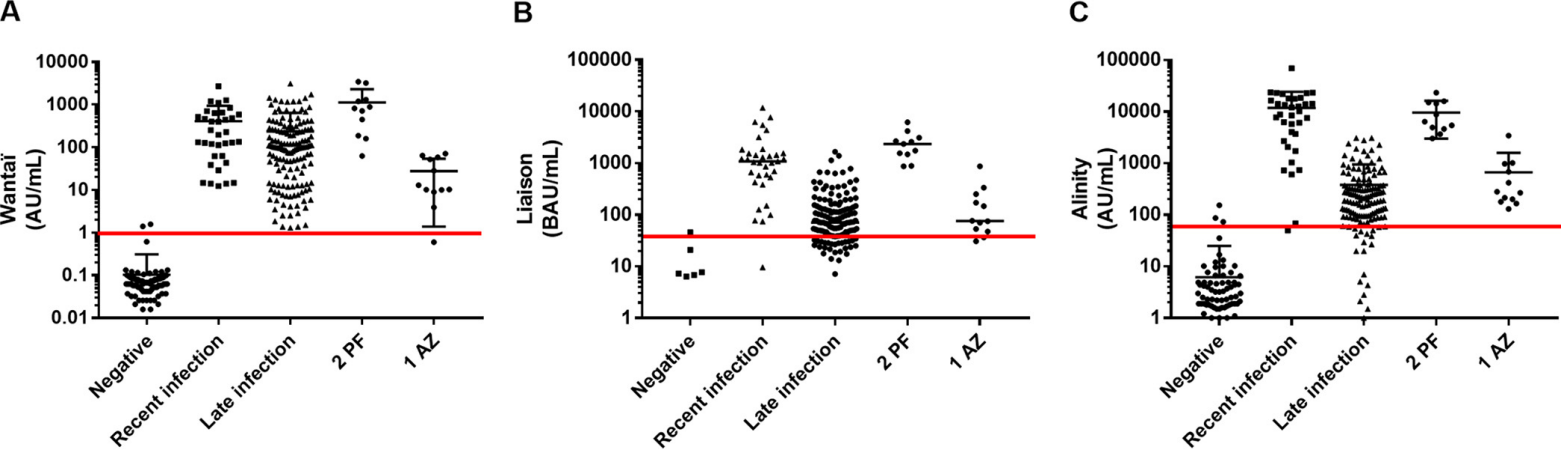
Distribution of the results. (A) Wantaï, (B) Liaison, and (C) Alinity assays according to patient groups. Black lines = median of each group. Red lines = manufacturer’s negative/positive threshold. Zero (0) values in the Liaison negative group (*n* = 92), the Liaison late infection group (*n* = 15), the Alinity negative group (*n* = 14), and the Alinity late infection group (*n* = 7) are not shown.

**FIG 2:**
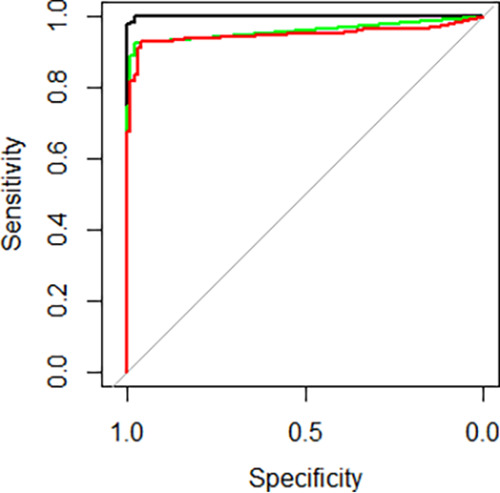
ROC curves for Wantaï (black line), Liaison (green line) and Alinity (red line). Gray line: y = x. The AUROCs were: Wantaï: 0.9996 (95% CI: 0.9403 to 0.9787), Liaison: 0.9475 (95% CI: 0.9208 to 0.9742) and Alinity: 0.9475 (95% CI: 0.9208 to 0.9742) indicating their capacity to accurately detect anti-SARS-CoV-2 antibodies.

All samples from prepandemic (*n* = 100), recent (*n* = 35) or late (*n* = 150) infected patients, and vaccinated people (Pfizer-BioNTech (PF), 2 doses, *n* = 11 and AstraZeneca (AZ), 1 dose, *n* = 12) were tested with the Wantaï, Liaison and Alinity assays. We recorded the qualitative (negative/positive) and quantitative (antibody titer) results for each sample (Fig. 1). The clinical specificities and sensitivities were calculated using the negative/positive threshold determined by the manufacturer. The specificities and sensitivities were 98%/100% (Wantaï), 99%/79.3% (Liaison), and 97%/88.9% (Alinity) (Fig. 1 and Table 1). Among negative samples, five were positive with only one assay and one was positive with two assays. The proportion of positive results in the “early” and “late” infection groups were 100%/100% (Wantaï), 97.1%/72.7% (Liaison), and 100%/84.7% (Alinity). It was 100% for all methods in the 2 dose PF vaccinated group and for Liaison and Alinity in the 1 dose AZ vaccinated group, and 91.7% in the 1 dose AZ vaccinated group for Wantaï (corresponding to 1/12 samples not detected) (Table 1). The Wantaï AUROC was significantly greater than those with Liaison (*P* < 0.001) or Alinity (*P* < 0.001). The Liaison and Alinity AUROCs were similar (*P* = 0.07) (Fig. 2).

**TABLE 1:** Immunoassay clinical performance using the manufacturers’ thresholds

Assay	Global performance			Subgroup sensitivity			
	AUROC (95% CI)	Specificity (95% CI) N = 100	Sensitivity (95% CI) N = 208	Early positive (95% CI) N = 35	Late positive (95% CI) N = 150	2 PF Positive (95% CI) N = 11	1 AZ Positive (95% CI) N = 12
Wantaï	0.9996 (0.9988–1)	0.98 (0.9256–0.9989)	0.9952 (0.9705–>0.9999)	1 (0.8824–1.000)	1 (0.970 to 1.000)	1 (0.6998–1.000)	0.9167 (0.6247–>0.9999)
Liaison	0.9595 (0.9403–0.9787)	0.99 (0.9401–>0.9999)	0.7933 (0.7329–0.8430)	0.9714 (0.8419->0.9999)	0.7267 (0.6501–0.7919)	1 (0.6998–1.000)	1 (0.7180 to 1.00)
Alinity	0.9475 (0.9208–0.9742)	0.97 (0.9117–0.9935)	0.8846 (0.8335–0.9218)	1 (0.8824–1.0000)	0.8467 (0.7798–0.8962)	1 (0.6998–1.000)	1 (0.7180 to 1.00)

We evaluated the agreement between the three assays by calculating Cohen’s kappa coefficient for each assay pair. The kappa values for Wantaï and Liaison (ĸ = 0.690 [95% CI: 0.611 to 0.768]), and for Wantaï and Alinity (ĸ = 0.798 [95% CI: 0.729 to 0.867]) indicated substantial agreement and almost perfect agreement between Liaison and Alinity (ĸ = 0.85 [95% CI: 0.792 to 0.909]).

### NIBSC standard and anti-SARS-CoV-2 antibody quantification.

We used serial 1:2 dilutions of the NIBSC standard to compare the results of the three immunoassays. We also determined the neutralizing antibody titers of each dilution with the live virus neutralization assay. Spearman’s rank coefficient tests showed that the results of each immunoassay and the reference anti-SARS-CoV-2 antibody concentration given by NIBSC standard dilutions (*r* = 1; *P* < 0.001 for each immunoassay/NIBSC pair) were linearly correlated as were those of our neutralization assay and the NIBSC antibody titers (*r* = 0.998; *P* < 0.001).

**FIG 3:**
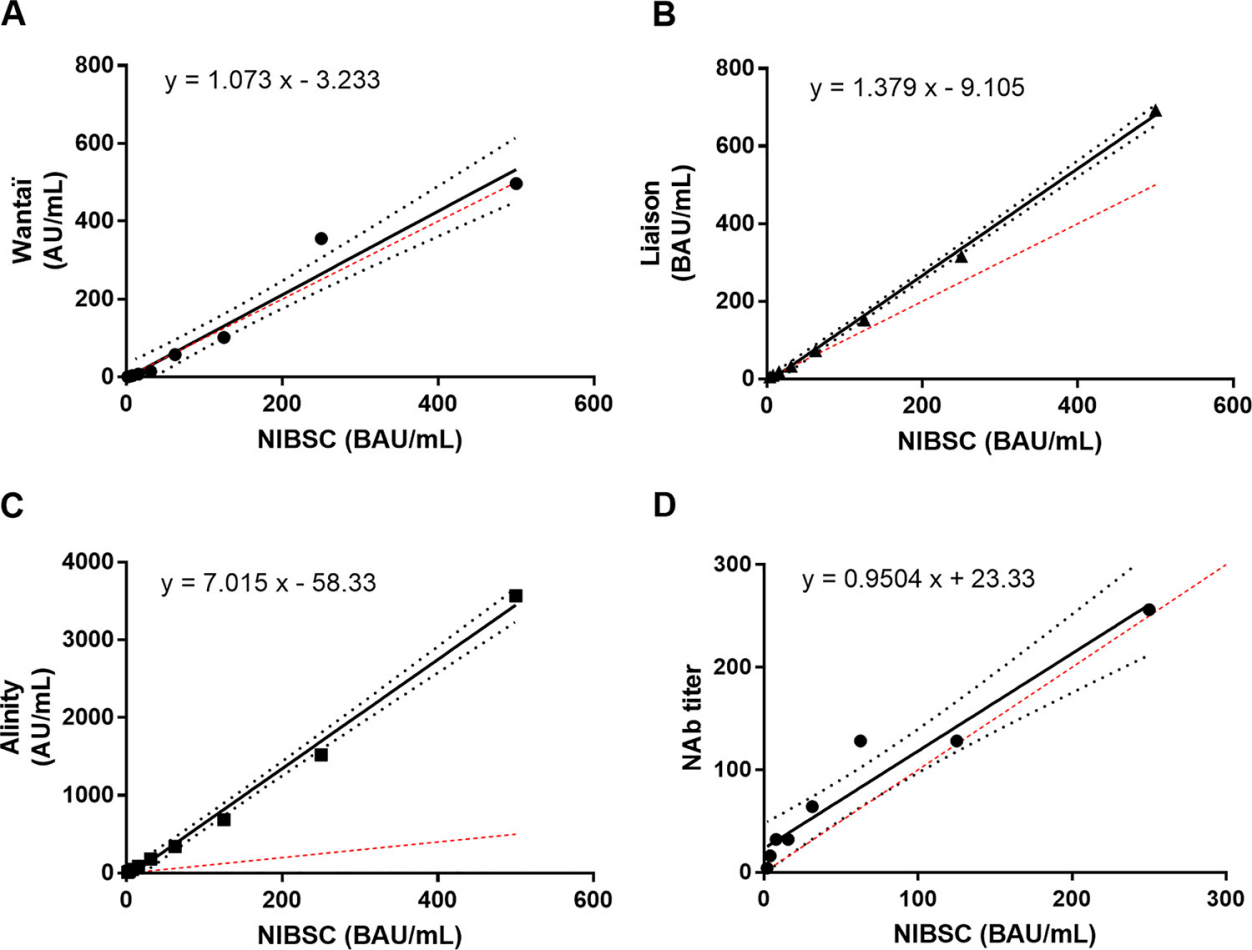
Quantification of anti-SARS-CoV-2 antibodies relative to the NIBSC international standard. Serial dilutions of the NIBSC 20/136 standard were assayed with the (A) Wantaï, (B) Liaison, and (C) Alinity assay. Neutralizing antibodies (NAb) were also determined with a live method (D). The black line represents the regression line and the dashed lines its 95% CI. The dashed red line represents the y = x line. AU: arbitrary units. BAU: binding antibody unit. The equations were y = 1.073 x − 3.233 (slope 95% CI: 0.8764 to 1.269; y-intercept 95% CI: −41.04 to 34.54) for Wantaï; y = 1.379 x − 9.105 (slope 95% CI: 1.314 to 1.443; y-intercept 95% CI: −22.27 to 4.057) for Liaison; y = 7.015 x - 58.33 (slope 95% CI: 6.501 to 7.529; y-intercept 95% CI: −157.3 to 40.61) for Alinity and y = 0.9504 x + 23.33 (slope 95% CI: 0.711 to 1.19; y-intercept 95% CI: −1.099 to 47.76) for NAb titers.

The calculated regression lines (*y*-axis: immunoassay Ab concentration or NAb titer; *x*-axis: NIBSC standard concentration) showed that the relations were linear and that the y-intercept 95% CI included the zero value, indicating that the y-intercept did not differ from 0 (Fig. 3A to D). The Wantaï assay slope did not differ from 1 (95% CI slope included 1), indicating concordance between Wantaï AU/mL and NIBSC BAU/mL. The proportional bias between Liaison and NIBSC BAU/mL was 1.3, while it was 7 between Alinity AU/mL and NIBSC BAU/mL. Finally, the slope of the neutralization assay was close to 1 (95% CI slope included 1), indicating concordance between NAb titers and NIBSC IU/mL (range: 1.9 to 250 IU/mL). The NIBSC concentrations giving a result close to the assay thresholds were: Wantai: 1.95 BAU/mL, Liaison: 31.25 BAU/mL, and Alinity: 7.81 BAU/mL.

### Correlation between the immunoassays’ quantitative values and neutralizing antibody titers.

**FIG 4:**
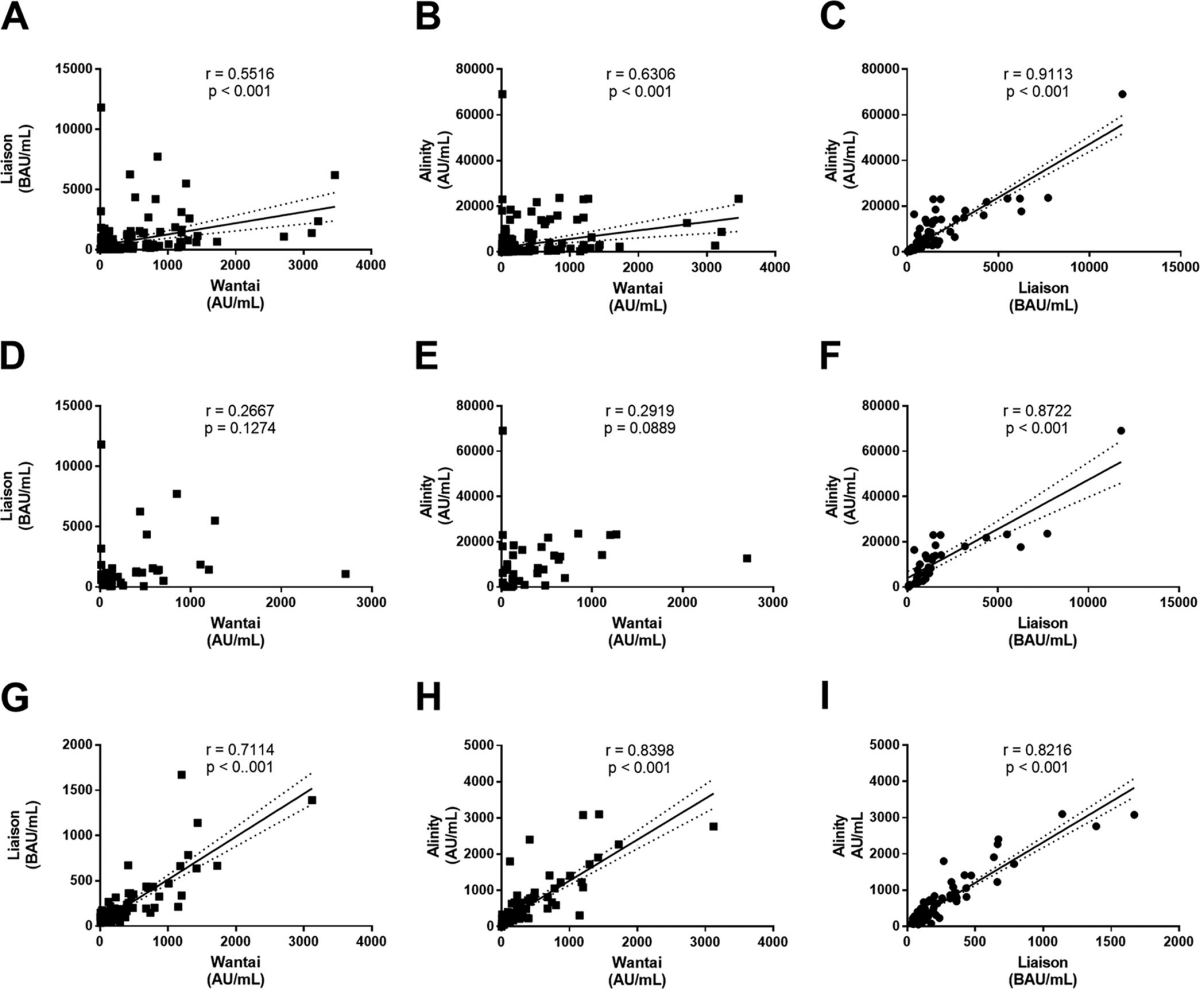
Correlation between the immunoassay results. Pairwise distribution of the Wantaï, Liaison, and Alinity assays values for all positive results (A to C), recent infections (D to F), and late infections (G to I). When the Spearman rank coefficient (r) indicated a significant correlation, the regression line was drawn. Dashed lines: 95% CI limits.

The median and interquartile range (IQR) for each patient group obtained with the immunoassays are shown in Table 2. We analyzed the pairwise correlation between the positive results obtained with the three methods. Spearman’s rank correlation test indicated that all pairwise correlations were positive and significant (*P* < 0.001) but that the R values for Wantaï/Liaison (*r* = 0.5516) and Wantaï/Alinity (*r* = 0.6306) were low (Fig. 4A to C). We also analyzed the correlations in the recent and late infection subgroups. Wantaï/Liaison and Wantaï/Alinity methods were not correlated when considering the recent infections (Fig. 4D to E), while Liaison/Alinity were (Fig. 4F). All pairwise correlations were significant in the late infection subgroup (Fig. 4G to I): regarding Liaison negative results, concentration ranges were 13 to 102 and 57.7 to 156.7 for the Wantai and Alinity assays, respectively, while concentration ranges were 1.3 to 53 and 46.6 for the Wantai and Liaison assays, respectively, in the Alinity negative results.

**TABLE 2:** Results: descriptive statistics

SARS-CoV-2 infection status	Wantaï	Liaison	Alinity	NAb^*a*^ titer
	Median [IQR] (Range)	Median [IQR] (Range)	Median [IQR] (Range)	Median [IQR] (Range)
Recent infection *n* = 35	195.3 [62.16–582.6] (12.33–2711)	1090 [531–1560] (9.65–11800)	8778.1 [2697–17723] (50.00–69093.2)	32 [32–64] (8–1024)
Late infection *n* = 150	97.77 [14.61–260] (1.30–3121)	71.1 [30.5–138.8] (0.00–1670)	186.3 [88.23–370.1] (0.00–3101.1)	32 [16–64] (2–256)
2 PF vaccinated *n* = 11	819.5 [187.4–1316] (62.63–3463)	2370 [1490–3130] (873–6190)	6402 [4608–15051] (3015.7–23319.2)	128 [64–128] (64–256)
1 AZ vaccinated *n* = 12	11.65 [9.275–56.93] (0.63–12.97)	76.2 [49–232.8] (30.9–870)	272.9 [183.9–905.9] (129.8–3430.1)	16 [8-52] (4–128)

^*a*^NAb, neutralizing antibody.

**FIG 5:**
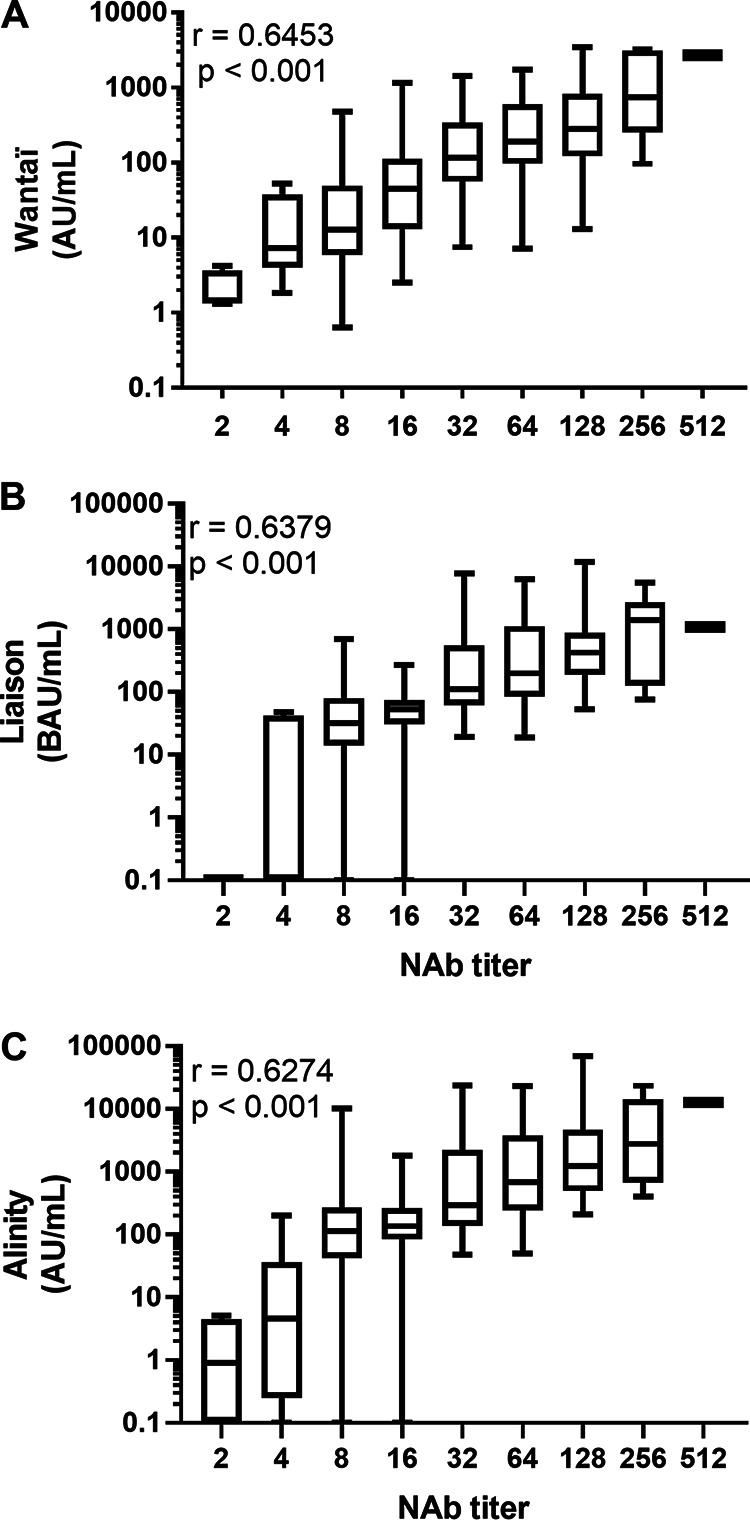
Immunoassays results and neutralizing antibody titers. Distribution of the Wantaï, Liaison, and Alinity assay values and the NAb titers for all positive results (A to C) The NAb titers were determined in a live virus neutralization assay using the B 1.160 strain. Spearman’s rank coefficients (r) and their *P* value are indicated. The box extends from the 25th to 75th percentiles and whiskers from minimal to maximal values.

We compared the results of each immunoassay with that of the titer obtained with a live virus neutralization assay. With the B.1.160 SARS-CoV-2 strain used in this assay, the Spearman rank coefficient *P* values for the relationship between the Wantaï, Liaison, and Alinity assay results and the neutralizing antibody titer indicated that the immunoassay results were positively correlated with the neutralizing antibody titer (Fig. 5A to C).

## DISCUSSION

We evaluated the clinical performance of three commercial high-throughput SARS-CoV-2 serological assays used in clinical diagnostics and the relationship between their quantitative results and the neutralizing antibody titer.

All three were highly specific, as previously reported for the Wantaï (16–18) and Alinity (19) assays. They accurately detected anti-SARS-CoV-2 antibodies in “early” sera, while the sensitivity of the Liaison and Alinity assays was lower for “late” sera. Other methods that detected antibodies directed against the S1 domain showed similar decreases in the IgG titer (20). The IgA concentration seems to be less affected (21, 22), which could contribute to the performance of the Wantaï assay because it detects total SARS-CoV-2 antibodies.

The quantitative values were pairwise correlated regardless of the tests compared but the correlation between Liaison and Alinity, which both detect IgG, was better. The Wantaï total Ig concentrations for the “early” infection group were not correlated with the Liaison or Alinity IgG concentrations. IgMs are detectable in “early” infections (12, 23), which could increase the total antibody concentrations and explain the observed lack of correlation. The relatively low sensitivity of Alinity and Liaison for “late” infection, comparatively to Wantai results, were observed with sera giving low concentrations with the Wantai assay.

The NIBSC international standard was also essential for comparing the antibody titers obtained with the commercial immunoassays (15). The lower detection limits determined with this standard for each assay agreed with the better diagnostic sensitivity observed with the Wantaï assay in the “late” infection group. While the immunoassay results and the NIBSC standard dilution values were significantly correlated, the Liaison and Alinity assays included proportional biases. Perkmann et al. (24), who analyzed the sera of 69 individuals sampled three weeks after their vaccination, reported a similar observation. The Liaison trimericS IgG assay results were correlated with those of other immunoassays, but the results were not interchangeable even when normalized to the NIBSC international standard (24). The bias of the Liaison assay (1.3) was lower than that of the Alinity assay (7) because the Liaison assay was calibrated using the NIBSC standard and results are expressed in binding antibody units, while those of the Alinity assay are given in arbitrary units. Interestingly, we observed a very good agreement between the arbitrary units used in the Wantai assay and the NIBSC concentrations in BAU/mL.

Finally, we found that antibody concentrations quantified by the immunoassays were positively correlated with the neutralizing antibody titers obtained in a live virus neutralization assay using the B 1.160 strain. Nonneutralizing antibodies, including anti-S antibodies, could promote the inflammation associated with COVID-19 via antibody-dependent cell cytotoxicity or cell-mediated immunity (25). However, the positive correlation we observed between immunoassays results and neutralization titers indicated that the former could be used to evaluate the neutralizing antibody response toward SARS-CoV-2 infection for a specific strain. The Spearman rank coefficients could be low because the live virus neutralization assay gave discontinuous values. Noval et al. (26) showed that the highest neutralizing sera correlate with detection of IgG, IgM, and IgA antibodies, while individuals with positive IgG alone had a poor neutralization response. Perhaps the Wantaï assay data were better correlated than those of the other immunoassays because IgMs and IgAs are more efficient neutralizers of SARS-CoV-2 than the IgGs (27).

The patients included in this work were infected at the beginning of the pandemic. Reduced sensitivity of new emerging SARS-CoV-2 to antibody neutralization has been reported (28). Preliminary data using our neutralization assay and the delta variant (B.1.617.2) indicate that the neutralizing antibody titers were reduced by a factor of 4. However, there was a good correlation between the neutralization tests. This point will have to be considered in the determination of the protective antibody titer.

A good correlation between antibody binding and neutralizing antibody titers could facilitate patient management and avoid time-consuming determination of neutralizing antibodies. We showed that the Wantaï total Ig concentrations and neutralizing antibodies titers of a population of health care workers were correlated and that a high neutralizing antibodies titer protected against reinfection (29). This correlation between neutralizing antibodies and anti-RBD-IgG determined by ELISA has also been found by others (30–32). It could be used to screen plasma donors to optimize the use of convalescent plasma to protect against lower respiratory tract disease. Mendrone-Junior et al. (32) screened convalescent plasma by determining the S/CO value of immunoassays corresponding to a NAb titer >160. Immunoassays are also essential for assessing the response to a vaccine in specific populations at risk of developing severe forms of COVID-19. Lastly, serological data will help to develop better care of immunocompromised patients, including additional boosters with standard or higher vaccine doses (33).

Biomedical laboratories are all equipped with multiparametric automates. The validation of commercial immunoassays to assess antibody response is a cost-effective approach while in-house live virus neutralization assay is time-consuming and needs dedicated infrastructure (L3 laboratory). Our evaluation of three quantitative immunoassays for determining neutralizing antibody titers in sera taken at different times after infection or vaccination has shown that they were not interchangeable, but could all be compared using the WHO international standard. Their results were significantly and positively correlated with the NAb titers, indicating that they will be extremely useful once the protective anti-SARS-CoV-2 antibody titer has been determined.

## MATERIALS AND METHODS

### Patient sera.

Anonymized blood samples were collected at the laboratory of Virology of the Toulouse University Hospital. Biobanking was done at the Center of Biological Resources of the Toulouse University Hospital (certified according to NF S96-900 standards). All French law ethical requirements were respected.

One hundred sera from patients hospitalized in January 2019 were used as anti-SARS-CoV-2 antibody-negative samples. The two panels of sera from infected patients represented “recent” and “late” infections. All SARS-CoV-2 infections were determined by a positive RT-PCR on a nasopharyngeal sample. Thirty-five recent samples were taken 15 to 35 days postinfection in patients hospitalized with COVID-19 (patients sampled between 1 April 2020 and 23 March 2021). Late samples (150) were taken more than 180 days postinfection in health care workers recovered from a SARS-CoV-2 infection (patients sampled 30 November to 4 December 2020). In this patient cohort, one-third were asymptomatic at the time of diagnosis of SARS-CoV-2 infection (34). We also established two groups of vaccinated subjects: one had been given two doses of the Comirnaty (Pfizer-BioNTech) vaccine, and the other had had one dose of the Vaxzevria (AstraZeneca) vaccine. Samples were collected at least 1 month after receiving the last dose.

The demographic characteristics of patients are listed in Table 3.

**TABLE 3:** Patient characteristics

SARS-CoV-2 Immunization status	No. of samples	Days from symptom onset	Sex M/F	Median age (yrs) (range)
Prepandemic	100	NA^*a*^	50/50	38.30 (0.57–82.26)
Recent infection	35	15–35	16/19	67.48 (26.33–95.26)
Late infection	150	>180	36/114	38.32 (21.15–73.35)
Vaccinated 2 PF^*b*^	11	NA	6/5	52.72 (27.4–66.19)
Vaccinated 1 AZ^*b*^	12	NA	4/8	43.06 (31.33–48.02)

^*a*^NA, not applicable.

^*b*^PF, Pfizer-BioNTech; AZ, AstraZeneca.

### NIBSC standard.

We used the first WHO International Standard for anti-SARS-CoV-2 immunoglobulin (human) as reference for anti-SARS-CoV-2 Ab titers (NIBSC code: 20/136, National Institute for Biological Standards and Control, Potters Bar, Hertfordshire, UK). This standard is supplied as a vial containing 250 IU for neutralizing antibody activity equivalent to 250 binding antibody units (BAU) for binding antibody assays. The lyophilisate was suspended in 250 µL ultrapure water and diluted in anti-SARS-CoV-2 Ab-negative serum.

### Immunoassays.

We evaluated three commercially available assays designed to quantify antibodies directed against the SARS-CoV-2 Spike (S) antigen. The Wantaï SARS-CoV-2 Ab ELISA (Beijing Wantaï Biological Pharmacy Enterprise Co., Ltd., Beijing, China) detects total anti-SARS-CoV-2 antibodies. ELISA plates were managed with the Bio-Rad EVOLIS device. Using positive samples, we determined a linear relationship between sample-to-cutoff (S/CO) and antibody concentration for samples in the 1.25 to 14.5 S/CO range. Samples with S/CO over 14.5 were diluted in phosphate-buffered saline containing 7.5% bovine serum albumin. We defined the S/CO ratio as arbitrary units/mL (AU/mL). The SARS-CoV-2 IgG II Quant for use with Alinity (Abbott Ireland, Diagnostics Division, Sligo, Ireland) and the LIAISON SARS-CoV-2 Trimeric S IgG (DiaSorin Inc., Stillwater, MN, USA) are designed to quantify anti-SARS-CoV-2 IgG. For each method, samples giving a result above the upper limit were diluted with the appropriate diluent, and the result was calculated. The manufacturers’ specifications are listed in Table 4.

**TABLE 4:** Assay manufacturer’s information^*a*^

Manufacturer	Assay name	Target antigen	Class of detected antibodies	Platform	Units	Quantification range	Manufacturer’s interpretation	Analytic specificity	Detection limit	Precision	Clinical specificity	Clinical sensitivity (>14 days post-positive RT-PCR or symptom onset)
Beijing Wantaï Biological Pharmacy Enterprise Co., Ltd.	Wantaï SARS-CoV-2 Ab ELISA	Spike RBD	Total	Manual ELISA *Automation on the EVOLIS plate manager (BIORAD)*	AU/mL	nd	<1: negative ≥1: positive	100%	nd	nd	100% (98.8–100%)	94.36 (90.87–96.81%) All onset time included
DiaSorin Inc.	LIAISON SARS-CoV-2 TrimericS IgG	Trimeric Spike	IgG	DiaSorin LIAISON XL Analyzer	BAU/mL	4.81-2,080	<33.8: negative ≥33.8: positive	99%	1.85 BAU/mL	3.9%–5.8%	99.5% (99.0–99.7%)	98.7% (94.5–99.6%)
Abbott Ireland Diagnostics Division	SARS-CoV-2 IgG II Quant	Spike RBD	IgG	Abbott Alinity I Analyzer	AU/mL	21-40,000	<50: negative ≥50: positive	100%	4.1 AU/mL	2.5%–6.1%	99.6% (99.22–99.80%)	99.35% (96.44–99.97%)

^*a*^AU, arbitrary units; BAU, binding antibody units; nd, not defined.

Specificity was calculated by the ratio of the number of prepandemic samples giving a negative result to the number of prepandemic samples (true negative/[true negative + false-positive]). Sensitivity was calculated by the ratio of the number of samples from infected patients giving a positive result to the number of infected patients’ samples [true positive/(true positive + false-negative)].

### Neutralization assay.

Neutralizing antibody titers were assessed using a live virus neutralization assay and a clinical SARS-CoV-2 strain (GISAID EPI_ISL_804378; GISAID Clade:GH; Pango lineage: B.1.160; Nextclade 20A.EU2) infecting Vero cells (ATCC, CCL-81). Briefly, 10^4^ cells were mixed with the virus suspension (100 50% tissue culture dose [TCID_50_]) and the tested serum and incubated for 4 days in 96-well plates. Two-fold serial dilutions (from 1:2 to 1:2048) of each serum were tested. The plates were examined to identify the wells showing a cytopathic effect (CPE). The titer was defined as the reciprocal of the highest serum dilution protecting cells from a CPE. The specificity of the neutralization assay evaluated on 100 sera from uninfected individuals was 100%. Its sensitivity was assessed in postinfection settings showing that 95.3% of sera with SARS-CoV-2 antibodies detected using the Wantaï ELISA contained neutralizing antibodies (34). Similarly, 99.5% of postvaccination sera with SARS-CoV-2 antibodies detected by ELISA contained neutralizing antibodies (35).

### Statistics.

IQR, means, 95% confidence intervals (CI), Cohen’s kappa coefficient, correlation, and regression analyses were determined using GraphPad Prism 7 (GraphPad Software, Inc.). AUROCs (areas under the receiving operator curve) were calculated and compared using the pROC package (36) in the R software (37).
